# The new era of artificial intelligence in neuroradiology: current research and promising tools

**DOI:** 10.1055/s-0044-1779486

**Published:** 2024-04-02

**Authors:** Fabíola Bezerra de Carvalho Macruz, Ana Luiza Mandetta Pettengil Dias, Celi Santos Andrade, Mariana Penteado Nucci, Carolina de Medeiros Rimkus, Leandro Tavares Lucato, Antônio José da Rocha, Felipe Campos Kitamura

**Affiliations:** 1 Universidade de São Paulo, Hospital das Clínicas, Departamento de Radiologia e Oncologia, Seção de Neurorradiologia, Faculdade de Medicina, São Paulo SP, Brazil.; 2 Rede D'Or São Luiz, Departamento de Radiologia e Diagnóstico por Imagem, São Paulo SP, Brazil.; 3 Universidade de São Paulo, Laboratório de Investigação Médica em Ressonância Magnética (LIM 44), São Paulo SP, Brazil.; 4 Academia Nacional de Medicina, Rio de Janeiro RJ, Brazil.; 5 Diagnósticos da América SA, São Paulo SP, Brazil.; 6 Centro de Diagnósticos Brasil, Alliança Group, São Paulo SP, Brazil.; 7 Grupo Fleury, São Paulo SP, Brazil.; 8 Universidade Federal de São Paulo, São Paulo SP, Brazil.

**Keywords:** Artificial Intelligence, Deep Learning, Machine Learning, Neuroradiology, Inteligência Artificial, Aprendizado Profundo, Aprendizado de Máquina, Neurorradiologia

## Abstract

Radiology has a number of characteristics that make it an especially suitable medical discipline for early artificial intelligence (AI) adoption. These include having a well-established digital workflow, standardized protocols for image storage, and numerous well-defined interpretive activities. The more than 200 commercial radiologic AI-based products recently approved by the Food and Drug Administration (FDA) to assist radiologists in a number of narrow image-analysis tasks such as image enhancement, workflow triage, and quantification, corroborate this observation. However, in order to leverage AI to boost efficacy and efficiency, and to overcome substantial obstacles to widespread successful clinical use of these products, radiologists should become familiarized with the emerging applications in their particular areas of expertise. In light of this, in this article we survey the existing literature on the application of AI-based techniques in neuroradiology, focusing on conditions such as vascular diseases, epilepsy, and demyelinating and neurodegenerative conditions. We also introduce some of the algorithms behind the applications, briefly discuss a few of the challenges of generalization in the use of AI models in neuroradiology, and skate over the most relevant commercially available solutions adopted in clinical practice. If well designed, AI algorithms have the potential to radically improve radiology, strengthening image analysis, enhancing the value of quantitative imaging techniques, and mitigating diagnostic errors.

## Resumo

A radiologia tem uma série de características que a torna uma disciplina médica especialmente adequada à adoção precoce da inteligência artificial (IA), incluindo um fluxo de trabalho digital bem estabelecido, protocolos padronizados para armazenamento de imagens e inúmeras atividades interpretativas bem definidas. Tal adequação é corroborada pelos mais de 200 produtos radiológicos comerciais baseados em IA recentemente aprovados pelo *Food and Drug Administration (FDA)* para auxiliar os radiologistas em uma série de tarefas restritas de análise de imagens, como quantificação, triagem de fluxo de trabalho e aprimoramento da qualidade das imagens. Entretanto, para o aumento da eficácia e eficiência da IA, além de uma utilização clínica bem-sucedida dos produtos que utilizam essa tecnologia, os radiologistas devem estar atualizados com as aplicações em suas áreas específicas de atuação. Assim, neste artigo, pesquisamos na literatura existente aplicações baseadas em IA em neurorradiologia, mais especificamente em condições como doenças vasculares, epilepsia, condições desmielinizantes e neurodegenerativas. Também abordamos os principais algoritmos por trás de tais aplicações, discutimos alguns dos desafios na generalização no uso desses modelos e introduzimos as soluções comercialmente disponíveis mais relevantes adotadas na prática clínica. Se cautelosamente desenvolvidos, os algoritmos de IA têm o potencial de melhorar radicalmente a radiologia, aperfeiçoando a análise de imagens, aumentando o valor das técnicas de imagem quantitativas e mitigando erros de diagnóstico.

## INTRODUCTION

Artificial intelligence (AI) has emerged as a promising tool in scientific research and medical healthcare. Machine learning (ML) and deep learning (DL) have offered means of evaluation of large datasets with intense computational developments that may provide considerate insights and new achievements in the diagnosis, prognosis, clinical, and surgical management of several neurological diseases. 1 2 3 Technographical reviews have demonstrated that the usage of technology may support, extend, and, to a lesser extent, replace some specific tasks performed by neuroradiologists. 4 It is of paramount importance to understand the potential contributions and limitations of AI in neuroradiology to embrace the available techniques and foster the development of useful tools that may ultimately impact clinical practice and patient management. Herein, our focus is to review the state-of-the-art AI translational research in the field of cerebrovascular diseases, demyelinating disorders, epilepsy, and neurodegenerative conditions.

## AI IN CEREBROVASCULAR DISEASES

Cerebrovascular diseases (CVDs) are a major cause of death and disability globally, frequently having devastating effects on patients and their families and a considerable negative impact on the healthcare system and the economy. 5 For the purpose of simplicity, we will adopt the clinical outcome-based classification of CVDs and divide them into hemorrhagic and ischemic conditions. Hemorrhagic diseases include arteriovenous malformations (AVM), intracranial aneurysms (IA), and intracranial hemorrhage (IH), whereas ischemic CVDs comprise atherosclerosis (AS), large vessel occlusion (LVO) / acute ischemic stroke (AIS) and Moya-moya disease (MMD). Although such diseases have different pathophysiological mechanisms, treatments, and prognoses, they all share a common characteristic: the diagnosis can be confirmed mainly using angiographic methods, whether CT angiography, MR angiography, or digital subtraction angiography (DSA).

The main objective of most works that use medical imaging is to extract semi-automatically or automatically features from an imaging modality that will be used in exploiting complex visual patterns associated with clinical outcomes and the classification at a study-, image- or pixel-level for a proper diagnosis. For this, rule-based algorithms or classification-based algorithms are used, the latter ranging from conventional machine learning ML algorithms to emerging deep learning DL algorithms. Although rule-based algorithm, such as Computer-Assisted Diagnostics (CAD) reflects a large portion of the various facets of AI for medical imaging, such tools have important constraints, such as the need for a detailed description of a set of rules detected by the human eye. An example is the identification of acute hemorrhage based on the establishment of an attenuation threshold above which pixels/voxels are selected. On the contrary, classification-based algorithms have the great advantage of identifying relevant features without requiring prior assumptions regarding their importance nor having to directly detail them, throughout the optimization of the error between the predicted and actual classification. 6

Although ML techniques represented a significant advancement over CAD, it was still challenging to accurately model complex imaging data due to the rigid analytic forms of traditional ML algorithms. In light of that, less than a decade ago, advances in computer hardware enabled DL, a class of highly adaptable algorithms. DL has emerged as the state-of-the-art ML method with specific characteristics, such as much greater potential for complexity and capacity to theoretically model any arbitrary mathematical function, that reflects greater generalization capacity and scalability, as well as superior performance.

**Figure 1:**
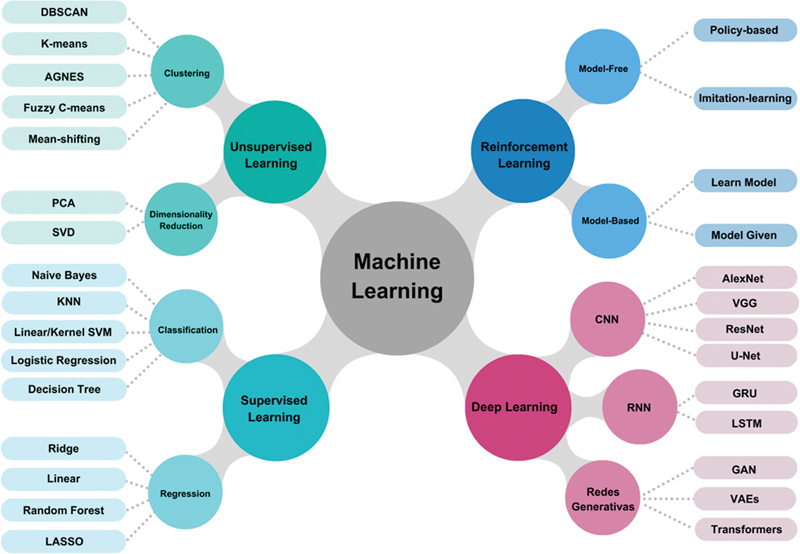
Framework of common machine learning algorithms.

An overview of the ML algorithms used in medical data analysis is given in ( Figure 1 ). Even though in the context of CVDs, conventional ML algorithms still seem to be more popular, as we move away from structured data - like that in electronic health records - to unstructured data -like that in medical imaging – DL potential to automatically extract underlying patterns directly from input images to predict target labels (without the need for feature vectors) gains relevance. 6 Regardless of the clinical claim, it is clear that DL solutions—whether Convolutional Neural Networks (CNN) or Recurrent Neural Networks (RNN)—are also heavily used in CVDs. On the one hand, the capability of CNNs to accept 2D or 3D images as input, in place of feature vectors, is its most novel feature. On the other hand, RNN and newer variations such as the long short-term memory network (LSTM) have achieved exceptional results for tasks requiring sequence labeling, in which time series/image order is important for the detection of an abnormality. Examples of such tasks in CVDs include diagnosis of diseases using MRI data composed of several images, consecutive frames in an ultrasound study (US), or multiple DSA frames with dynamic flow changes between them. 7

**Figure 2:**
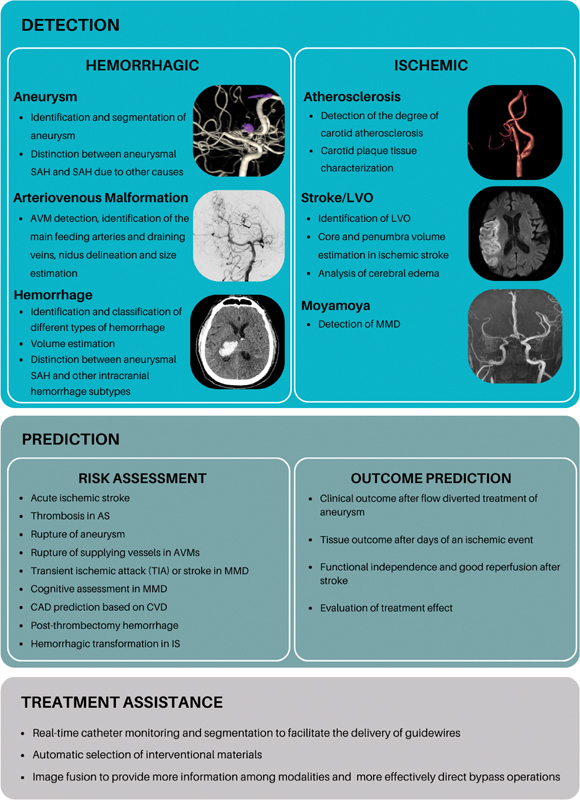
Abbreviations: SAH, subarachnoid hemorrhage; AVM, arteriovenous malformation; LVO, large vessel occlusion; MMD, Moya-moya disease; AS, atherosclerosis; TIA, transiente ischemic attack; CAD, coronary artery disease; CVD, cerebrovascular disease; IS, ischemic stroke. Artificial Intelligence based applications in cerebrovascular diseases.

In the field of CVDs, AI approaches are primarily divided into detection, prediction (which mainly includes risk assessment and outcome prediction), and treatment assistance ( Figure 2 ). Detection tasks include the location and segmentation of aneurysms, or identification and volume estimation of subtypes of acute hemorrhage. 5 8 Prediction tasks generally include risk prediction and outcome estimation. An example of the former is the identification of high-risk patients for cerebral or retinal ischemic events based on clinical characteristics and ultrasonic plaque features. 7 Examples of the latter are the prediction of the number/volume of lesions on DWI and T2/FLAIR days after an ischemic event based on the acute images, and prediction of functional independence (modified Rankin Scale ≤2) and good reperfusion (post-mTICI ≥2b) at 3 months after stroke using baseline clinical variables associated with treatment variables. 9 10 For risk and outcome prediction tasks to perform well, as opposed to detection (which typically rely on a single modality as input), a variety of data must be integrated as input, including imaging findings with clinical, demographic, morphological, or hemodynamic characteristics. This favors the use of more complex models and architectures comprised of multiple integrated algorithms, that is, fusion models and multimodal AI. Finally, examples of treatment-aid tasks include real-time catheter monitoring and segmentation to facilitate greatly the delivery of guidewires later on or image fusion to more effectively direct bypass operations. 5

### Hemorrhagic cerebrovascular diseases

Besides IH itself, the two hemorrhagic CVDs that have likely benefited the most from AI are aneurysm and AVM. Identifying these diseases using imaging scans is crucial for patient management since 10-20% of nontraumatic subarachnoid hemorrhage (SAH) are fatal and mainly caused by aneurysm and AVM rupture. 11

Concerning aneurysms, up to 2015, the great majority of papers for automatic detection used ruled-based or ML algorithms such as support vector machine (SVM), logistic regression (LR), Naïve Bayes, or random forest (RF). 12 13 From 2017 on, studies focused on the use of DL models such as Res-Net, VGG, and U-Nets to identify and segment aneurysms, rather than in CTA, TOF MRA images, 2D-DSA or 3D rotational DSA (3DRA). 14 15 16 17 In addition to the detection and segmentation, other classifiers combined image data with clinical, morphological, and hemodynamic information (e.g. wall shear stress, oscillatory shear) to predict individual rupture status of unruptured intracranial aneurysms and clinical outcome 6 months after flow diverted treatment. 18 19 20 CNNs have also been used to distinguish aneurysmal subarachnoid hemorrhages from other intracranial hemorrhage subtypes. Finally, ML techniques have been adopted to predict the outcome of aneurysmal SAH and the outcome after the rupture of the anterior communicating artery aneurysm. 21 22

Compared with aneurysms, very few studies on AVM have been published, although AVM *nidus* are not hard to detect. A number of studies combine various imaging approaches and modalities for AVM segmentation and assessment. They vary from center-line extraction methods (such as skeletonization approach) to unsupervised ML classifiers applied on CTA images and DL models applied on MRI images in order to differentiate, the *nidus* from brain tissue, and avoid unnecessary exposure of the healthy parenchyma to radiation during radiosurgery. 23 24 25 In an interesting study, a system was built to assist AVM diagnosis. 26 For that, a faster RCNN was trained on 2D-DSA videos to track the appearance order of important vascular structures from the videos and quantify them as temporal features. Then, an SVM classifier combined this information with morphological features (obtained from radiomic methods applied in key frames) for AVM diagnosis and grading system.

Although 2D-DSA is still the “gold standard” for the diagnosis of AVM, new techniques, such as 3DRA and 4D-CTA have demonstrated similar value for AVM detection, identification of the main feeding and drain vessels, *nidus* delineation, and size estimation. 27 28 Following Garcia et al.'s results, these modalities may be more thoroughly studied in AI models for AVM segmentation in the upcoming years. 29

Diverging a bit from morphological features, a study evidenced the robustness of quantitative DSA (QDSA) features of a selected ROI (e.g. peak density and time to peak) to predict the rupture of the AVM nidus. 30 For further prediction tasks, such as the prediction of post-operative results, the usage of other traditional methods is also discussed. 31

Finally, computer systems to assist treatment in CVDs have been researched far less than for detection and prediction, since they usually require hardware implementation. Examples of these were introduced in a study to segment and track catheters on 2D X-ray sequences during endovascular aneurysm repair, and in other studies that introduced some novel conceptions of treatment in hemorrhagic CVDs, including automatic selection of interventional materials and real-time guidance in surgery. 5 32

### Ischemic cerebrovascular diseases

Detection of large vessel occlusion (LVO) and AIS are two of the most pursuable objectives in applying AI to cerebrovascular pathology. Over the past decade, 13 companies have launched automated and semi-automated commercially available software for acute stroke and LVO diagnostics (Aidoc®, Apollo Medical Imaging Technology®, Brainomix®, inferVISION®, RAPID®, JLK Inspection®, Max-Q AI®, Nico.lab®, Olea Medical®, Qure.ai®, Viz.ai®, and Zebra Medical Vision®). 33 Also, considerable research work has been reported with the aid of ML models, mostly using medical images as input. While CT images have been used mainly for hemorrhage identification, LVO detection, and automated ASPECT calculation, MRI images have been used primarily for automatic core volume estimation on DWI images, evaluation of penumbra, and to predict the final ischemic stroke lesions from initial MRI, stroke symptom onset and hemorrhagic transformation. 34 35 36 37 38 39 40 41 AI has also been used to improve image quality and speed acquisition - since time delays associated with brain scanning is a real constrain – for stroke risk prediction, for analysis of cerebral edema, and to evaluate treatment effect. 42 43 44

Except for stroke, to the best of our knowledge, a small number of essays discuss the computer-assisted analysis of other ischemic CVDs such AS and MMD, and for these abnormalities, models tend not to use medical pictures as input. This might be because the diagnostic criteria for AS, for instance, are much more complex and less standardized than the morphological or gray-level attributes used to identify aneurysms. Examples are ML algorithms trained to detect the degree of carotid atherosclerosis (CAS) using clinical indicators, including age, gender, hypertension, cholesterol, and glucose levels, as well as pulse wave features. 45 46 Similarly, ML and DL were used in a dataset of 636 subjects, to diagnose AS while relying solely on a few clinical criteria and reaching an accuracy of 96.4%. 47

Regarding carotid plaque tissue characterization, a systematic review evidenced that US imaging was the most commonly used imaging technique for applying AI methods (94.59%), followed by CT (30%) and MRI (35%). 7 Most articles used manual and statistical analysis, followed by ML (especially SVM, RF, and DT). In addition, the most common architecture used in the DL framework for all three imaging modalities was U-Net. Although DL is a modality that is still emerging, it might be promising to address some of the challenges of plaque evaluation, since it can characterize plaque's composition in spite of variations and fuzziness in pixel distribution to be non-linear. In fact, a CNN was proposed to classify plaque components into lipid core, fibrous cap, and calcified tissue and achieved a correlation coefficient of 0.90 between automatic measurement and expert measurement. 48 Finally, since CVDs cover both stroke and myocardial infarction, carotid intraplaque neovascularization in B-mode ultrasonography have been applied to multiclass ML to predict coronary artery disease (CAD). 49 Such an automated system is likely to become a prominent CAD detection system in the future.

Lastly, various medical imaging modalities have been used in pertinent DL-based applications for MMD automatic detection, including plain skull radiography, T2-weighted MRI and DSA images. 50 51 52 DL and sparse representation-based classification were also used to predict the hemorrhagic risk of MMD in DSA and to assess vascular cognitive impairment on resting-state functional MRI, respectively. 53 54

## AI IN EPILEPSY

Epilepsy affects nearly 70 million people around the world, with a huge social and economic burden. 55 Adequate epilepsy care usually requires a multidisciplinary work-up team that seeks a precise diagnosis, comprehension of cognitive comorbidities and impact on quality of life, response to pharmacological treatment, and decisions regarding surgical management and prognosis. It is also important to bear in mind that epilepsy may be caused by distinct underlying diseases, such as hippocampal sclerosis, malformations of cortical development, infectious diseases, autoimmune conditions, brain scars, and tumors. 56 While positron emission tomography (PET), single photon emission computed tomography (SPECT), and magnetoencephalography may help to localize cerebral dysfunction, magnetic resonance imaging (MRI) may identify and accurately delineate underlying specific pathologies, some of them amenable to surgical treatment. However, many unanswered questions remain in the debate of epilepsy care, such as cryptogenic seizures, the involvement of otherwise apparently normal brain parenchyma, and complex networks between epileptogenic foci and diverse cognitive deficits.

A combination of statistics, data science, and computational resources may yield big-data problem-solving and shed light on a more comprehensive approach to several neurological conditions. Therefore, a collaboration of scientists in multicentric studies has generated advancements in global neuroscience, such as the Enhancing NeuroImaging Genetics through Meta-Analysis (ENIGMA) Consortium. 56 The ENIGMA-Epilepsy working group has applied advanced methods such as structural covariance and event-based-modeling, combined with DL approaches to analyze large datasets of structural MRI, diffusion tensor imaging (DTI), and resting state functional MRI of patients with epilepsy and healthy controls. 57 This group recently demonstrated that multivariate ML approaches could successfully classify healthy controls and patients with temporal lobe epilepsy (TLE) secondary to hippocampal sclerosis (HS), as well as to lateralize the side of TLE, using morphological features from T1-weighted data and diffusion MRI parameters. 58

Another intriguing study showed that NeuroQuant, an FDA-approved automated software, had lower sensitivity and negative predictive values but had specificity and positive predictive values comparable to neuroradiologists' visual assessments in the detection of HS in a large cohort of TLE patients undergoing presurgical evaluation. 59 Although studies so far do not demonstrate that quantitative imaging can replace visual MRI assessment by experienced neuroradiologists in the detection of HS, there may be a role for the use of these tools in the preliminary evaluation of surgical candidates in centers that lack such professionals.

Malformations of cortical development (MCD) account for the most common surgical pathologies in children and the third most common cause in adults with pharmacoresistant focal epilepsies. 60 Out of them, focal cortical dysplasia represents the vast majority, and its diagnosis heavily depends on both the skill of the examiners and the optimization of MRI protocols, which are preferentially acquired using high-field strength scanners. 61 Post-processing tools show promise for assisting in the detection of more subtle lesions, but some cases still remain overlooked. 62 Recently, one multicentric study validated the use of a DL algorithm to detect MRI-negative FCD with multimodal MRI data. 63

It is well known that seizures are not the sole component of epilepsy that affects the quality of life of patients and their families. Affected individuals may also suffer from deficits in several cognitive domains, including language and memory, and some studies using ML algorithms have accurately predicted cognitive comorbidities. 64 65 Interestingly, AI has been used to predict brain age in patients with epilepsy. These studies revealed that patients had significantly older brain ages than healthy controls, and functional brain age was related to decreased cognition capabilities. 66 67

It seems that ML and DL algorithms might also be useful in the prediction of clinical prognosis after surgical treatment of medically refractory epilepsy. Previous works that incorporated quantitative volumetric MRI measurements demonstrated that subtle cortical brain atrophy beyond the surgical site influences seizure-free outcomes, reinforcing the idea that focal epilepsies are complex network disorders. 68 69 In addition, DL methods were applied to structural connectomes to predict postsurgical seizure outcomes at least one year after epilepsy surgery. 70

Despite the emerging AI tools in epilepsy, future works are needed to clarify if these applications are not only a transient hype and if they will ultimately prove to be useful in care at an individual end-user level.

## AI IN MULTIPLE SCLEROSIS

MRI has a crucial role in the diagnosis and monitoring of multiple sclerosis (MS) patients. The definition of MS according to the McDonald criteria requires the clinical and/or MRI identification of central nervous system (CNS) lesions disseminated in time (DIT) and space (DIS). 71 However, with the recent definition of “new” demyelinating entities, such as neuromyelitis optica spectrum disorders (NMOSD) and myelin-oligodendrocyte glycoprotein antibody-associated disease (MOGAD), there is an increasing need for a more precise description of lesion shapes and the use of new MRI biomarkers with a better representation of the pathological substrate of MS, such as central vein sign, cortical lesions and paramagnetic rim lesions. 72 73 74 75

The primary goal in the follow-up and control of MS patients is to achieve the status of no evidence of disease activity (NEDA), which essentially consists of the absence of clinical symptoms or progression, new or expanding T2-FLAIR demyelinating lesions, and no new T1-gadolinium enhanced lesions. 76 This highlights the need for a precise lesion count in MRI readings. Atrophy measurements are also becoming increasingly important in the MRI follow-up of MS patients as the relevance of the degenerative processes for the course of the disease is recognized. 76 However, in everyday practice, several of these measurements, including lesion count and analysis of changes in lesion volumes, might be very time-consuming. Manual measurements of the brain and spinal cord volume can take even more time and might also be imprecise and with low reproducibility. Artificial intelligence techniques could therefore be helpful for a faster and more precise quantification and follow-up of MS patients.

In the last few years, the use of AI in MS to foresee diagnosis, forecast long-term prognosis and ensure trustworthy findings and time efficiency has received a lot of attention in recent years. The use of AI in medical imaging, particularly MRI, has shown encouraging results, enabling automated lesion and tissue segmentation, disease categorization, and contract synthetization from advanced sequences. Such a strategy is also appropriate for the emerging field of "omics," where the evaluation of enormous data sets gathered from a single patient is essential from the standpoint of personalized medicine. 77

AI can be used to interpret and analyze MR images of patients with MS, detecting subtle changes in the images of central nervous system structures, including the brain and spinal cord, over time. ML algorithms can be trained on large MRI image datasets to recognize specific patterns and features associated with MS. These algorithms can identify brain and spinal cord lesions characteristic of MS, such as demyelinating plaques, with high precision and speed, as well as track the emergence of new lesions and the increase in lesion volume by analyzing time series of MR images. 78

New advances in the automation of combined methods, the traditional unsupervised machine learning technique, and the DL attention-gate 3D U-net network have improved the detection of MRI lesions in infratentorial and juxtacortical regions. 79 ML algorithms are trained using clinical, imaging, and genetic data to identify patterns and characteristics that are indicative of disease in order to make predictions or decisions. DL can be applied to analyze MR images at a deeper level, identifying subtle and complex features of the MS-related lesions and MRI normal-appearing tissues. A great advantage of DL is the ability to handle large amounts of complex, unstructured data, such as high-resolution medical images. However, training deep neural networks usually requires a large set of annotated data and considerable computational resources. 80 81

The central vein sign (CVS) has emerged as a promising MRI biomarker to improve the accuracy and speed the diagnosis of MS. The introduction of the CVS concept has added a new dimension to the diagnostic capabilities of MRI in MS, distinguishing MS lesions from non-MS white matter lesions that mimic its clinical and radiological features. The presence of a central vein within an MS lesion is thought to reflect the underlying pathological process of MS, which involves inflammation and destruction of the myelin surrounding the veins. 74 The veins appear as dark signal voids on T2-weighted or T2*-weighted MRI sequences due to the flow-void effect. 82 The CVSnet, a DL-based prototype for automated evaluation of the CVS in white matter MS lesions, was tested using data from several hospitals and has since allowed for bigger multicenter trials to determine the merit of including the CVS marker in MS diagnostic criteria. 83

There are several commercial volumetric MRI analysis solutions that can assist in lesion identification and segmentation by providing automatized, quantitative, and qualitative measurements for the diagnosis and monitoring of MS. Among others, CorTechs.ai, Icometrix, Qynapse, Pixyl are examples of such tools that received regulatory approval and have helped with clinical decision-making and the development of personalized therapies. 84

Another promising application of AI in MS diagnosis is the analysis of data from sensors and wearable devices. These devices can collect a variety of data such as movement, heart rate, and sleep quality. AI algorithms can process this data and identify patterns that could be related to MS symptoms such as fatigue, balance issues, and gait changes. This information can be useful both for the diagnosis and ongoing monitoring of patients with MS. 80 85

Although some of these tools are validated in compelling databases, it is still important to understand their impact in the clinical setting in different populations. Furthermore, it is intuitive that a faster and more precise diagnostic might be provided by AI, supposedly having a positive economic impact on healthcare. 86 However, the use of AI also implies additional costs and more and wider studies of the economic impact of the use of AI in MS are needed.

In summary, AI offers useful data that speed up the identification and measurement of targeted lesions as well as the measurement of atrophy and degenerative processes. It is crucial to emphasize though, that AI is only a supporting tool; medical professionals are still ultimately responsible for the diagnosis, and the final interpretation of MRI scans. Clinical experience and medical judgment are still essential for medical care and to guide the future of AI. 87

## AI IN NEURODEGENERATIVE DISEASES

Neurodegenerative diseases involve different clinical conditions with various pathological substrates that, in general, result in significant disability with major social and economic impact. The diverse group of neurodegenerative diseases, which have shown an increase in prevalence with population aging, includes dementia syndromes such as Alzheimer's disease (AD), movement disorders like Parkinson's disease (PD) and atypical parkinsonism, as well as Amyotrophic Lateral Sclerosis (ALS). 88

Since the clinical manifestations of many of these disorders overlap and a firm anatomopathological diagnosis is impractical, diagnosing them might be difficult. It is not uncommon for the proper diagnosis to be delayed, which affects the prognosis. In such instances, neuroimaging has gained significant relevance, particularly structural MRI and functional Nuclear Medicine. 89

The main role of MRI is to exclude other causes that might justify the symptoms, such as sequelae of vascular/traumatic events, tumors, hydrocephalus, among others. 90 Moreover, the development of MRI sequences and high-field MRI scanners like the 3T has led to novel imaging discoveries that have been supportive in either ruling out or confirming clinical hypotheses.

In addition, AI advances, whether through quantification methods or DL techniques, will be essential for assessing neurodegenerative diseases in a number of ways, including detecting the onset of the disease, characterizing it, aiding in differential diagnoses, quantifying disease progression, and evaluating treatment response.

### Dementia syndromes

Evaluation of dementia syndromes by structural magnetic resonance allows for identifying atrophy patterns that predict assertive diagnosis. Volumetric T1-weighted sequence plays a fundamental role, providing adequate spatial resolution and cortical atrophy pattern characterization. However, due to the lack of specific findings, specific diagnosis of some dementia faces some resistance, and incipient imaging findings are frequently neglected. 90 In this context, it was mandatory to develop systematic and practical analysis, with assessment scales, aiming for interpersonal uniformity. For example, the evaluation of medial temporal atrophy in AD through Scheltens scale and, more recently, through the visual scale described by Urs et al. 91

Visual scales limitations are not neglectable, and automatic measurement and stratification systems have been improved. Although some platforms are expensive to use, they can deliver rapid results. Through quantification techniques, supported by AI and the OASIS database, some studies have shown up to 96.85% accuracy in detecting AD compared to healthy controls, 84.3% in differentiating between AD and frontotemporal dementia, and 97.48% in differentiating between AD and mild cognitive decline. 92

### Parkinson's disease and other movement disorders

The role of MRI has grown in importance over the past few years in the setting of PD and its differential diagnosis, particularly Progressive Supranuclear Palsy (PSP) and Multiple Systems Atrophy (MSA), despite not being included in the Movement Disorder Society diagnostic criteria. 93

With the advent of the 3T and high-resolution sequences, in 2014 the loss of the *swallow tail* sign was depicted, with high specificity and sensitivity for the identification of parkinsonism. 94 It reflects the loss of susceptibility sequence hypersignal in dorsolateral substantia nigra pars compacta (nigrosome-1 topography) due to probable iron deposits and neuromelanin reduction. Despite being considered a biomarker of PD, the loss of the swallow sign is not a diagnostic criterion, as it is found in atypical parkinsonism. However, the negative predictive value of 100% suggests that the presence of the sign makes the diagnosis of PD much less likely.

Furthermore, the evaluation of neuromelanin in the substantia nigra, through magnetization transfer T1-weighted sequences (T1-MTC), is also considered a biomarker of PD, useful even in the evaluation of progression. 95 A 2022 study demonstrated that neuromelanin quantification is progressively reduced among patients with isolated REM sleep behavior disorder (considered a prodromal stage of PD) and patients with established PD compared to healthy controls. 47 In addition, DL was used to delineate automatically the regions of interest in the substantia nigra pars compacta (SNc) and demonstrated comparable results to manual measurement, but with the advantage of being faster. 96

Despite advances in AI research in PD, its use is still limited due to several constraints, including small samples, low accuracy, and limitations specific to the ML algorithms. 97 MRI also allows the evaluation of other PSP and MSA imaging findings, such as brainstem morphometric rates and depiction of pontine hot cross bun sign and putaminal slit sign. 93

Finally, studies using a support vector machine (SVM) demonstrated accuracy, specificity, and sensitivity of 91%, 88%, and 93%, respectively, in distinguishing MSA and PD patients. Another study that similarly used SVM models achieved an accuracy of 91.7% for MSA detection using mesencephalic evaluation. 92

### Amyotrophic lateral sclerosis

ALS is a neurodegenerative disease that affects upper and lower motor neurons. Its prognosis is quite limited, with life expectancy ranging between 3 to 5 years after the onset of symptoms. 98 The diagnosis of lower motor neuron involvement is based on electroneuromyography and muscle biopsy. In contrast, the diagnosis of upper motor neuron involvement is challenging and the role of MRI must be highlighted. In 2012, T1-MTC hyperintensity along the corticospinal tract (CST) was described, demonstrating high specificity (100%), despite low sensitivity (37%). 99

Advances in AI in ALS have so far focused on the diagnosis, mainly on the differential diagnosis with other neurodegenerative diseases that also affect motor function, such as PD, Huntington's disease, and MSA. Also, AI has been useful in understanding disease pathological processes. A recent study demonstrated an accuracy of 70-94% for distinguishing four clinical/radiological phenotypes of ALS patients (with signal alteration on CTS, without signal alteration on CST, classic patients with obvious lower motor neuron and upper motor neuron clinical signs, and patients with frontotemporal dementia) using a Random Forest (RF) algorithm. 100 Another study also showed an accuracy of 78.7% in the estimation of patient survival, mainly in the short survival group, through the use of MR sequences (DTI combined with T1-weighted images) and DL. 98

What we recognize is that, in addition to the advancement of neuroimaging, especially with regard to technical sequences evolution, analysis, and report standardization, the complete evaluation of neurodegenerative patients is essential for diagnosis or follow-up. Future projections in the set of neurodegenerative diseases management aim at earlier diagnosis, in prodromal phases, so that specific therapies, still under development, may impact on disease's natural history, before neuronal loss and consolidated disability. Additionally, it has been argued that AI may help in providing precision, agility, and even solutions to a number of unresolved questions.

In conclusion, AI exhibits significant potential for advancing the field of neuroimaging, offering transformative solutions for both diagnostic and therapeutic applications. Despite this promise, the integration of AI algorithms into clinical practice is currently confined to a limited number of specialized research institutions. This limitation is principally attributed to two critical barriers:

- the lack of algorithmic generalizability across diverse datasets and;
- the absence of sustainable business models for widespread adoption.

As technological innovations continue to address these challenges, it is anticipated that an increasing number of AI-based models will transition from experimental stages to clinical implementation. Another crucial aspect of AI in medicine is that even an exemplary AI model is not a guarantee for improved clinical care outcomes. Both under-reliance and over-reliance on AI algorithms can compromise their efficacy, either by failing to capitalize on their diagnostic or therapeutic capabilities, or by diminishing the role of human clinical expertise in medical decision-making, respectively. The way clinicians engage with AI algorithms critically influences the potential for either substantial benefits or detriments in healthcare outcomes.
